# Mysterious bio-duck sound attributed to the Antarctic minke whale (*Balaenoptera bonaerensis*)

**DOI:** 10.1098/rsbl.2014.0175

**Published:** 2014-04

**Authors:** Denise Risch, Nicholas J. Gales, Jason Gedamke, Lars Kindermann, Douglas P. Nowacek, Andrew J. Read, Ursula Siebert, Ilse C. Van Opzeeland, Sofie M. Van Parijs, Ari S. Friedlaender

**Affiliations:** 1 Integrated Statistics, 172 Shearwater Way, Falmouth, MA 02540, USA; 2 Australian Antarctic Division, 203 Channel Highway, Kingston, Tasmania 7050, Australia; 3 NOAA Fisheries Office of Science and Technology, 1315 East-West Highway, Silver Spring, MD 20910, USA; 4 Alfred Wegener Institute for Polar and Marine Research, Am Handelshafen 12, 27570 Bremerhaven, Germany; 5 Duke University Marine Laboratory, 135 Pivers Island Road, Beaufort, NC 28516, USA; 6 Institute for Terrestrial and Aquatic Wildlife Research, University of Veterinary Medicine Hannover, Foundation, Werftstrasse 6, 25761 Büsum, Germany; 7 NOAA Northeast Fisheries Science Center, 166 Water St., Woods Hole, MA 02543, USA; 8 Marine Mammal Institute, Hatfield Marine Science Center, Oregon State University, 2030 Marine Science Drive, Newport, OR 97365, USA

**Keywords:** *Balaenoptera bonaerensis*, bio-duck, Antarctic minke whale, Southern Ocean, acoustic monitoring

## Abstract

For decades, the bio-duck sound has been recorded in the Southern Ocean, but the animal producing it has remained a mystery. Heard mainly during austral winter in the Southern Ocean, this ubiquitous sound has been recorded in Antarctic waters and contemporaneously off the Australian west coast. Here, we present conclusive evidence that the bio-duck sound is produced by Antarctic minke whales (*Balaenoptera bonaerensis*). We analysed data from multi-sensor acoustic recording tags that included intense bio-duck sounds as well as singular downsweeps that have previously been attributed to this species. This finding allows the interpretation of a wealth of long-term acoustic recordings for this previously acoustically concealed species, which will improve our understanding of the distribution, abundance and behaviour of Antarctic minke whales. This is critical information for a species that inhabits a difficult to access sea-ice environment that is changing rapidly in some regions and has been the subject of contentious lethal sampling efforts and ongoing international legal action.

## 1. Introduction

The bio-duck sound has been recorded ubiquitously in the Southern Ocean by researchers for over five decades. First described and named by submarine personnel in the 1960s, the bio-duck has since been recorded at various locations in the Southern Ocean, but its source remained a mystery [1–6]. The sound consists of a regular series of downswept pulses, ranging from 50 to 300 Hz, with harmonics of up to 1 kHz. The number of pulses within a series can differ within and between recording locations, but the sound is highly repetitive with a typical interval of 3.1 s between the start of two series [1]. The enigma surrounding the sound has been further deepened by its discordant seasonal occurrence patterns. During winter and spring, the bio-duck occurs simultaneously in the eastern Weddell Sea and off Western Australia, indicating a very widespread distribution of the species, or potentially a seasonal migration by one segment of the population and year-round presence in Antarctic waters by another [3,6].

Here, we present conclusive evidence attributing the bio-duck sound to Antarctic minke whales. We describe acoustic recordings from multi-sensor acoustic recording tag (Acousonde) deployments on two Antarctic minke whales in Wilhelmina Bay, Antarctic Peninsula. These were the first acoustic tags deployed on Antarctic minke whales, providing a unique opportunity for detailed study of their vocalizations.

## 2. Material and methods

In the austral summer (13 and 15 February) 2013, two Antarctic minke whales were tagged with multi-sensor suction-cup tags, equipped with an HTI-96-MIN hydrophone (High Tech, Inc., Long Beach, MS, USA; sensitivity: −187.2 dB re 1 V µPa^−1^), recording continuously at a sample rate (SR) of 25811 Hz. The recording system had a flat frequency response (±3 dB) in the 22–9292 Hz frequency band. In addition to acoustic data, auxiliary sensors sampled temperature, pressure, 3-axis accelerometry and magnetometry at 10 Hz. Tags were deployed in Wilhelmina Bay, off the western Antarctic Peninsula (64°41′ S, 62°13′ W and 64°38′ S, 62°16′ W) using a hand-held carbon fibre pole from a rigid-hulled inflatable boat (RHIB).

Spectrograms (fast Fourier transform (FFT) size: 4096 points, 95% overlap, Hanning window, time and frequency resolution: 8 ms and 6 Hz) were generated and analysed using Raven Pro v. 1.5 [7]. Presence of vocalizations was evaluated manually based on these spectrograms, and start and end time (s), 90%-energy duration (s), peak, centre and first and third quartile frequencies (Hz) were measured for each identified sound. Vocalizations were filtered between 22 and 200 Hz using a 4-pole Butterworth bandpass filter, and RMS received levels (RLs) were calculated within the 90% duration time window using MATLAB (2007a, The MathWorks Inc., Natick, MA, USA).

In addition, vocalizations were compared to example data from PALAOA (22 July 2006; 70°31′ S, 8°13′ W) [8]; Dumont D'Urville (3 June 2006; 65°33′ S, 140°32′ E) [9] and Ross Island (22 November 1964; 77°30′ S, 168°00′ E) [10].

## 3. Results

**Figure 1.**
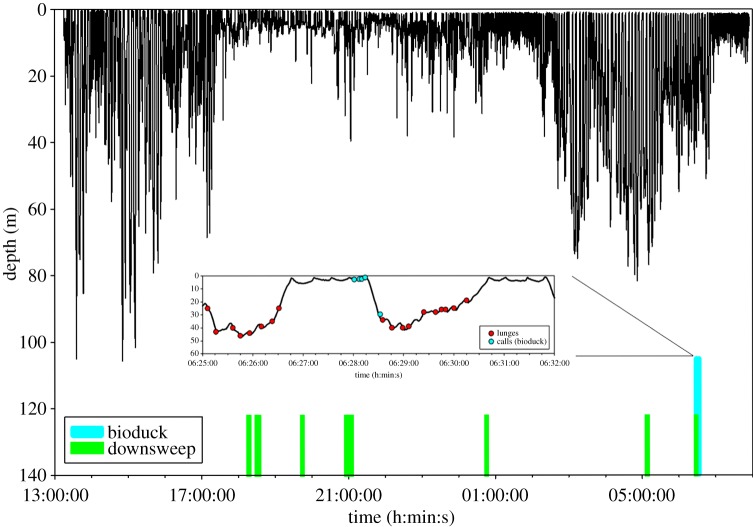
Complete dive profile of the Antarctic minke whale tagged in Wilhelmina Bay (64°41′ S, 62°13′ W) on 13/14 February 2013. Times at which vocalizations occurred are marked with vertical bars (green, downsweep; turquoise, bio-duck sound). Inset shows detail of two lunge-feeding dives (lunges marked by red circles) during which bio-duck sounds were recorded on the tag.

**Figure 2.**
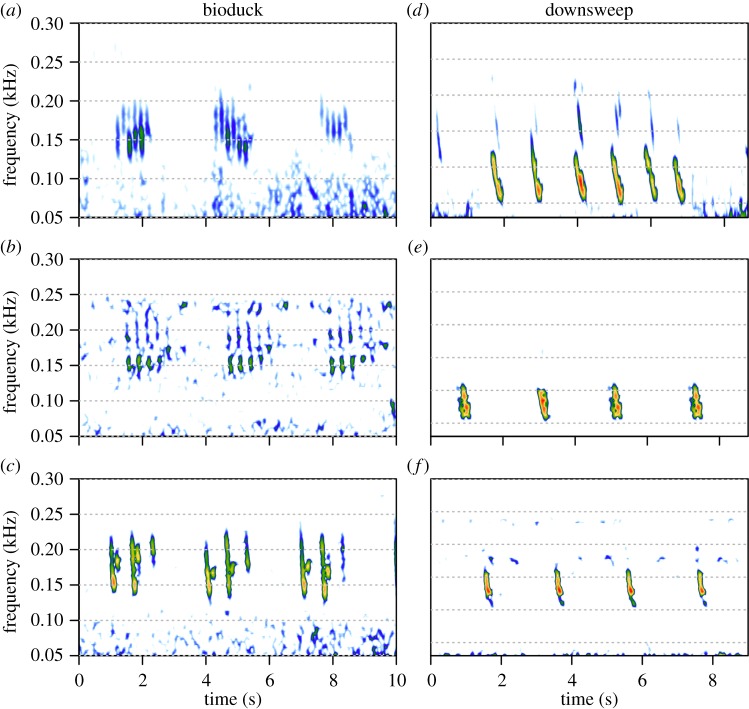
Bio-duck and downsweep sounds compared between different recording locations. *Bio-duck*: (*a*) Wilhelmina Bay (14 February 2013; 64°41′ S, 62°13′ W; acoustic recording tag; SR: 25 811 Hz; filtered and downsampled to 2000 Hz; FFT: 512; 95% overlap; Hanning window); (*b*) PALAOA station (22 July 2006; 70°31′ S, 8°13′ W; long-term recorder; SR: 48 000 Hz; filtered and downsampled to 2000 Hz; FFT: 512; 95% overlap; Hanning window); (*c*) Dumont D'Urville (3 June 2006; 65°33′ S, 140°32′ E; long-term recorder; SR: 4000 Hz; filtered and downsampled to 2000 Hz; FFT: 512; 95% overlap; Hanning window). *Downsweeps*: (*d*) Wilhelmina Bay (13 February 2013; 64°41′ S, 62°13′ W; acoustic recording tag; SR: 25811 Hz; FFT: 4096; 95% overlap; Hanning window); (*e*) Ross Island (22 November 1964; 77°30′ S, 168°00′ E; opportunistic recording; SR: 2000 Hz; FFT: 512; 95% overlap; Hanning window); (*f*) PALAOA station (22 July 2006; 70°31′ S, 8°13′ W; long-term recorder; SR: 48 000 Hz; filtered and downsampled to 2000 Hz; FFT: 512; 95% overlap; Hanning window) (see the electronic supplemental material for all sound files). (Online version in colour.)

The two tags recorded for 18 and 8 h, respectively. During both deployments the tagged whales were in large single-species groups of five to about 40 animals and fed almost continuously [11]. Vocalization rates were low; only 32 clear calls, with a signal-to-noise ratio of more than 10 dB, were recorded in this entire dataset. Most calls (*n* = 26) were recorded when the tagged animal was close to the surface (mean ± s.d.: 2.6 ± 0.7 m). The bio-duck sound (*n* = 6) was recorded on one of the tags, just before a feeding dive (figure 1). The vocalization consisted of series of 5–12 pulses, produced in regular sequences at an interval of 3.1 s (measured from the start of one series to the start of the next). Most energy was contained between 146 ± 12 and 165 ± 16 Hz (mean ± s.d., first and third quartiles), and pulses exhibited peak frequencies of 154 ± 13 Hz. The 90% energy duration of individual pulses was 0.1 s. The identification of these sounds as the bio-duck was based on comparisons with the published literature [1,3–6]. In addition, based on spectral and temporal content, tag recordings were matched to bio-duck sounds recorded on long-term, bottom-mounted recorders at PALAOA [8] (70°31′ S; 8°13′ W) and at Dumont D'Urville [9] (65°33′ S; 140°32′ E) (figure 2*a*–*c*). Comparisons with the PALAOA recordings in particular revealed similarity in frequency range, number of pulses, and in the stereotypic interval of 3.1 s between bio-duck series (figure 2*a*,*b*).

Apart from the bio-duck sound, low-frequency downsweeps (*n* = 26) were the most frequently recorded sound on both tags, with a mean peak frequency of 83.1 ± 16.7 Hz, and a duration of 0.2 s (figure 2*d*–*f*). Low-frequency downsweeps (60–130 Hz) have previously been recorded in the Ross Sea (77°30′ S 168°00′ E) during a close encounter with two Antarctic minke whales [10]. These sounds have very similar characteristics to our data (figure 2*d*,*e*). In addition, similar downsweeps were recorded in conjunction with the bio-duck sound at PALAOA (figure 2*f*) and in Western Australia [1].

Bio-duck RLs at the tag averaged 140.2 ± 3.6 dB re 1 μPa, and downsweeps were received at a mean RL of 147.3 ± 5.3 dB re 1 μPa (table 1). One complication of acoustic tag recordings is the difficulty in ascribing calls to the focal animal [12]. However, during daylight, tagged whales were visually tracked from a RHIB. During these focal follows, no other marine mammal species were observed within 1 km of the focal minke whale groups. Previous calculations of source levels for minke whale vocalizations were in the range of 160–165 dB re 1 μPa [13,14]. Given the reported RLs, assuming spherical spreading (20 × log(*R*)) [15] and source levels for the bio-duck sound to be similar to those reported for other minke whale sounds, the sound source was within one to two body lengths of the recording tag. Given the observation of large groups in which animals were frequently associated, the absence of other species during the time when calls were recorded and RLs that indicate a source close to the tagged animals, we conclude that recorded sounds were produced by either the focal animal or other Antarctic minke whales in the immediate vicinity.

**Table 1.** Acoustic parameters (mean ± s.d.) of bio-duck (*N* = 6/*n* = 41 pulses) and downsweep (*N* = 26) sounds recorded on two acoustic recording tags. *n*(P), number of individual pulses; PF, peak frequency; CF, centre frequency; Q25, first quartile frequency (25%); Q75, third quartile frequency (75%); DUR90(P), 90% energy duration of individual pulses/downsweeps; RMS RL, RMS received level.

	*n*(P)	PF (Hz)	CF (Hz)	Q25 (Hz)	Q75 (Hz)	DUR90 (P) (s)	RMS RL (dB re 1 *μ*Pa)
bio-duck	7 ± 3	154 ± 13	155 ± 13	146 ± 12	165 ± 16	0.1 ± 0.0	140.2 ± 3.6
downsweep	—	83 ± 17	84 ± 17	75 ± 15	94 ± 15	0.2 ± 0.1	147.3 ± 5.3

## 4. Discussion

This study is the first to analyse acoustic tag recordings from Antarctic minke whales. Our results solve the mystery around the source of the bio-duck sound, which is one of the most prevalent sounds in the Southern Ocean during austral winter and can now be attributed unequivocally to the Antarctic minke whale. These results have important implications for our understanding of this species, which is of particular priority to the International Whaling Commission [16,17].

Antarctic minke whales live in remote open-water environments and within sea ice habitats [18]. Traditional ship-based study methods are extremely expensive, and data from such studies are complex and difficult to interpret [19–21]. The acoustic identification of Antarctic minke whales offers the opportunity to retrospectively analyse several years’ worth of existing long-term recordings to explore seasonal occurrence and migration patterns of this species, including the possibility of using acoustics to estimate abundance [22]. Of particular interest in this respect is the prevalence of the bio-duck in Antarctic waters during austral winter [6], indicating that a large part of the population may stay in ice-covered waters year-round. Similar results have been suggested from visual sighting records [23,24]. However, recordings of the sound off Western Australia also during winter indicate that while one population segment remains in the ice, part of the population may undertake seasonal migrations to lower latitudes [3]. A reduced occurrence of the bio-duck sound in Antarctic summer recordings [6] probably relates to a change in behaviour and reduced vocal activity during times of intense foraging [11] as suggested by the low call rates in our recordings, rather than a change in the relative abundance of whales during this time.

Acoustic recordings can provide insight into potential population differentiation based on geographical differences in vocal behaviour. For example, bio-duck sounds from Dumont D'Urville, East Antarctica [9], as well as sounds reported in archived recordings made in the Ross Sea [2], exhibited three pulses per burst. In contrast, recordings of bio-duck sounds from West Antarctica [8], including the sounds described here, typically have five to six pulses.

In conclusion, the identification of the Antarctic minke whale as the source of the bio-duck sound will allow a more detailed understanding of the behavioural ecology of this abundant, but poorly understood species. Furthermore, the value of passive acoustic monitoring will be significantly increased in remote areas of the Antarctic, especially during austral winter, when visual surveys are essentially infeasible.
